# BayesianKAN: A reaction condition optimization framework integrating Kolmogorov‐Arnold network and Bayesian optimization

**DOI:** 10.1002/smo2.70082

**Published:** 2026-08-05

**Authors:** Juntao Wang, Yujing Zhao, Peiyu Yi, Liyuan Duan, Qingwei Meng, Qilei Liu

**Affiliations:** ^1^ State Key Laboratory of Fine Chemicals Department of Pharmaceutical Sciences Institute of Chemical Process Systems Engineering School of Chemical Engineering Dalian University of Technology Dalian China; ^2^ MOE Key Laboratory of Bio‐Intelligent Manufacturing School of Bioengineering Dalian University of Technology Dalian China; ^3^ Ningbo Institute of Dalian University of Technology Ningbo China

**Keywords:** Bayesian optimization, Kolmogorov‐Arnold network, machine learning, reaction condition optimization, symbolic regression

## Abstract

Efficient optimization of chemical reaction conditions is crucial for enhancing reaction yield and selectivity, yet traditional methods face inherent limitations including experimental inefficiency, low predictive accuracy, and poor interpretability. This study proposes a novel framework for reaction condition optimization by integrating the Kolmogorov‐Arnold network (KAN) model and Bayesian optimization (BO) algorithm. The KAN model establishes accurate and explicit mappings between reaction conditions and outcomes like yield, while BO iteratively optimizes reaction outcomes to identify optimal conditions based on the KAN model, demonstrating efficacy even with sparse data. This framework is implemented as the BayesianKAN software and validated in two reaction systems: hydrogen peroxide (H_2_O_2_) synthesis and photocatalytic acceptorless dehydrogenation to flavones. KAN exhibits superior fitting accuracy and generalization ability compared to the traditional response surface methodology and other seven common machine learning approaches. Experiments also verify the feasibility and effectiveness of BayesianKAN, achieving 60.7% and 5.2% increases in H_2_O_2_ production and flavone yield, respectively.

## Graphical abstract

A novel BayesianKAN framework integrates Kolmogorov‐Arnold networks with Bayesian optimization to efficiently navigate complex factor spaces, accelerating the discovery of optimal reaction conditions for chemical synthesis.

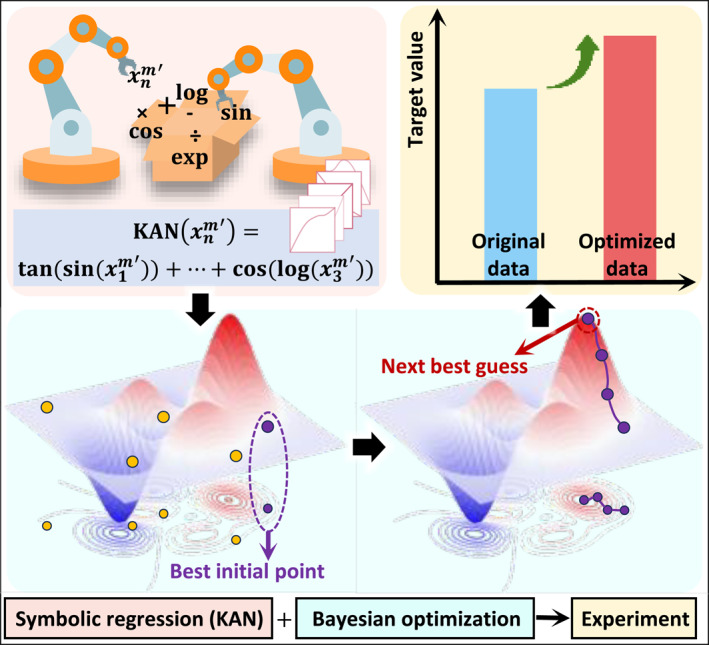

## 1 INTRODUCTION

The optimization of reaction conditions in chemical synthesis is pivotal for enhancing yield and selectivity, as this process not only affects the purity and yield of the final product but also determines the economic feasibility and sustainability of the entire synthetic procedure. In traditional experiments, the one factor at a time (OFAT) approach is extensively employed, characterized by the independent investigation of each variable to observe the specific effects of individual factors on the reaction.[^1^, ^2^] However, this approach overlooks the potential interactions between multiple variables to some extent. Furthermore, the repeated experimentation required by OFAT often consumes extensive time and resources, limiting its applicability to complex reaction systems. To address these limitations, the design of experiments (DoE) approach garners increasing attention. This approach enables the comprehensive evaluation of multiple factors within a defined range and enhances research efficiency to some extent through systematic experimental design.[^3^, ^4^, ^5^] Nevertheless, while DoE demonstrates certain advantages in multivariable analysis, the results often struggle to capture the complex nonlinear relationships between reaction conditions and product yield/selectivity. Additionally, when experiments involve high‐dimensional factors, substantial numbers of experiments are still required to identify the optimal reaction condition, leading to challenges in reducing time and resource consumption.

To further enhance the efficiency of experimental design, the integration of DoE with response surface methodology (RSM) is proposed. For example, Mane et al.^[6]^ combine DoE with RSM to optimize the reaction conditions for palladium‐catalyzed biphenyl synthesis, under which the maximum biphenyl yield of 83% is achieved, accompanied by a 35% reduction in catalyst concentration compared to the initial conditions. This approach, through RSM‐based modeling, effectively reduces the required number of DoE‐based trials while demonstrating a certain level of predictive capability in describing the quantitative relationships among conditions. Studies show that RSM is able to flexibly fit experimental data using linear or nonlinear models, thereby capturing interactions among multiple factors to some extent.[^7^, ^8^] However, existing nonlinear models are typically limited to quadratic models, which exhibit insufficient fitting capabilities in complex reaction systems and struggle to fully characterize the intricate relationships within high‐dimensional factor spaces.

To address the aforementioned challenges, machine learning methods have garnered significant attention. For instance, Gaussian process (GP) models are frequently employed to fit nonlinear relationships between experimental conditions and outcomes, serving as surrogate models for Bayesian optimization (BO) algorithms to achieve reaction condition optimization.^[9]^ By leveraging the probabilistic models constructed through GP, BO iteratively screens promising experimental conditions to guide subsequent experiments. This characteristic enables BO to efficiently explore a wide factor space even under limited resources and constrained experimental trials.[^10^, ^11^] For instance, Sugisawa et al.^[12]^ employ BO to optimize the reaction conditions for the rapid and mild one‐flow synthesis of unsymmetrical sulfamides. The findings demonstrate that optimal reaction conditions affording yields ≥75% are successfully identified after only 10 experimental runs. Furthermore, the preeminent role of BO in navigating complex, high‐dimensional chemical reaction spaces has been underscored by the comprehensive review by Desimpel et al.,^[13]^ together with the experimental studies by Düker et al.^[14]^ and Fuse et al.^[15]^ Among its components, the GP serves as the core tool of BO, capable of accurately capturing the complex relationships between experimental conditions and reaction performance.[^16^, ^17^] However, GP exhibits limited interpretability, making it difficult to understand the internal mechanisms of the model, especially in complex reaction systems.

In summary, conventional approaches for optimizing chemical reaction conditions exhibit inherent limitations. Early OFAT fails to account for interaction effects among multiple factors. While DoE has enhanced multivariate analysis capabilities, it still suffers from inefficiencies in experimental exploration. RSM improves efficiency by reducing the number of experiments, yet its reliance on simple polynomial models limits predictive accuracy in complex nonlinear relationships. Although the GP model in BO exhibits excellent nonlinear fitting abilities, it is hindered by poor interpretability, which obstructs the understanding of factor interaction mechanisms. Such interpretability is critical in chemical engineering context, serving not only as a quantitative prerequisite for validating reaction mechanisms but also as a cornerstone for translating optimization strategies into industrial scale‐up. To address these challenges, this study proposes a novel framework (BayesianKAN) for reaction condition optimization by coupling the Kolmogorov‐Arnold network (KAN) model with BO. Notably, the KAN model holds significant promise for the physical and chemical sciences, owing to its robust generalization performance even with limited samples and high explicit interpretability.[^18^, ^19^, ^20^, ^21^] These advantages have been preliminarily demonstrated in domains such as materials discovery,^[22]^ quantum chemistry,^[23]^ and biochemical interactions.^[24]^ In Section 2, the workflow of the proposed framework is described in detail, including the data preparation, KAN model, BO algorithm, and experimental verification. In Section 3, two case studies involving the hydrogen peroxide (H_2_O_2_) synthesis and photocatalytic acceptorless dehydrogenation to flavones are presented to highlight the feasibility and effectiveness of the proposed framework in optimizing reaction conditions.

## 2 BAYESIANKAN FRAMEWORK FOR REACTION CONDITION OPTIMIZATION

**FIGURE 1:**
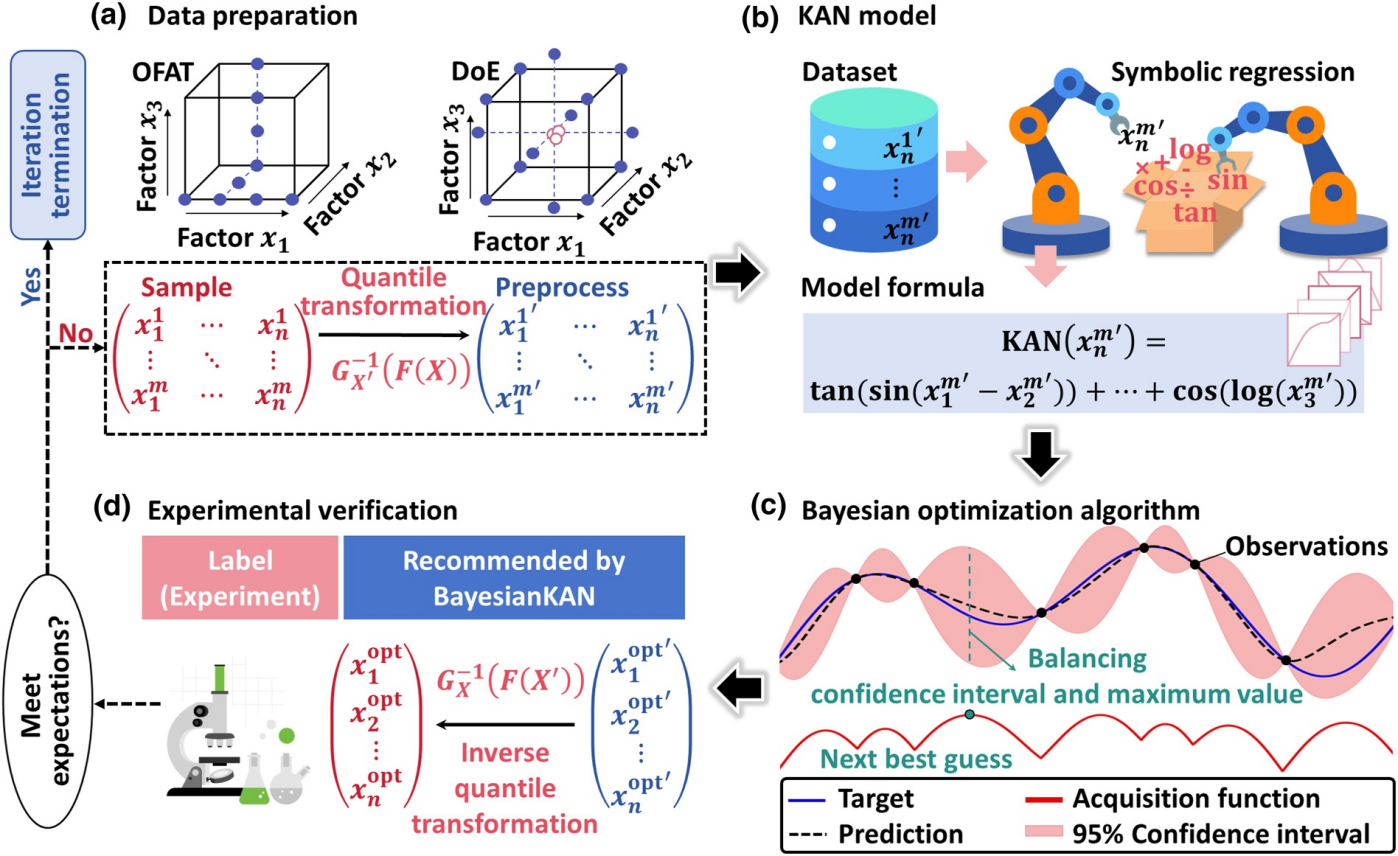
An overview of the BayesianKAN framework for reaction condition optimization.

An overview of the BayesianKAN framework is briefly described in Figure 1. First, the experimental data of a certain number of samples is prepared through either OFAT or DoE approach, and the values of sampled reaction conditions are processed using the quantile transformation method to enhance the robustness and predictive performance of the subsequent KAN model (Figure 1a). Next, the processed reaction conditions are fitted with experimental reaction outcomes (reaction labels) (e.g., yield) by the KAN model, which serves as the target function for BO (Figure 1b). Then, the BO algorithm is used to maximize reaction outcomes based on KAN predictions (Figure 1c). During this process, the optimal reaction conditions for the next experimental trial are recommended. Finally, the recommended reaction conditions are validated by experiments to obtain experimental reaction outcomes (Figure 1d). The experimental results are subsequently utilized to retrain the KAN model, which is further employed in conjunction with the BO algorithm to recommend a new set of reaction conditions. This iterative process is repeated until the experimental outcomes meet the desired criteria.

### 2.1 Data preparation

Experimental data preparation represents foundational steps in constructing the BayesianKAN framework. In this study, data preparation is carried out using either OFAT or DoE, with the DoE sampling methods including three‐level full factorial design, central composite design (CCD), and Box‐Behnken design.^[5]^ Additionally, data preprocessing is performed using the quantile transformation method.

**FIGURE 2:**
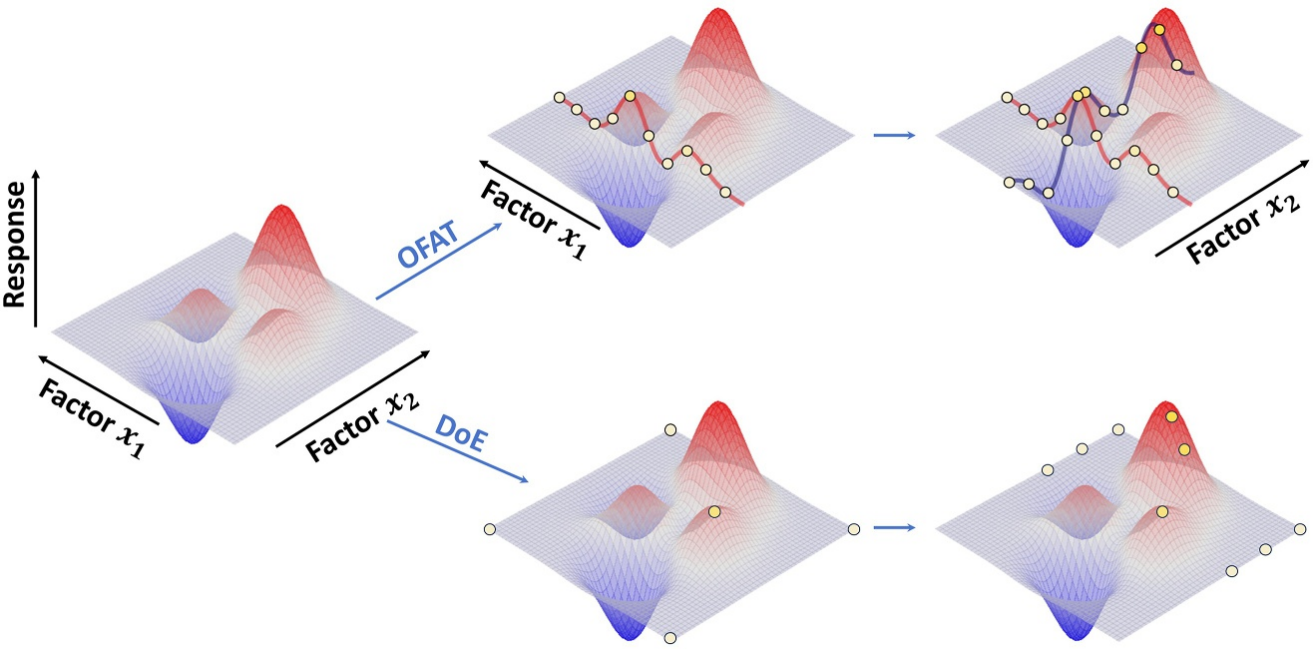
Data preparation (OFAT and DoE).

**Sample reaction conditions:** Reaction conditions are first sampled using either OFAT or DoE. In the OFAT approach (Figure 2), experiments are conducted by fixing all but one factor per trial and sampling that single factor. Upon identifying its optimal value, this factor is fixed, and the process iterates sequentially through the remaining factors until all reach their determined optima.[^2^, ^25^] Alongside the OFAT method, DoE (Figure 2) serves as another sampling strategy in this study. DoE is a class of statistical methods that aims to mathematically describe the output of a process based on multiple simultaneously varied inputs. Recognized as a robust technique, DoE facilitates sampling that not only ensures combination uniformity but also comprehensively accounts for factor interactions. Although OFAT sampling inherently suffers from non‐uniform data distribution compared to DoE, it remains the predominant approach in most real‐world experimental designs. Consequently, this study integrates OFAT‐derived datasets into the BayesianKAN framework to enhance its practical relevance. The feasibility and effectiveness of using OFAT samples for BayesianKAN are subsequently validated through the H_2_O_2_ synthesis case study presented in the following section.

**Preprocess reaction conditions:** To enhance the stability and predictive performance of the subsequent KAN model, the sampled data is further preprocessed by applying quantile transformation to the reaction conditions.^[26]^ This method is particularly suitable for small sample sizes, as it effectively maps the original data to a uniform or standard normal distribution.[^27^, ^28^] The quantile transformation method not only mitigates issues related to uneven variable distributions but also strengthens model robustness and generalization ability.^[29]^

Quantile transformation is a data preprocessing method based on probability distribution mapping. Its mathematical definitions (Equations (1) and (2)) are as follows, including formulas for quantile transformation and inverse transformation.

(1) $$\begin{array}{c} X^{\prime }=G_{X^{\prime }}^{-1}\left(\int_{-\infty }^{x}f_{X}\left(t\right)dt\right) \end{array}$$

(2) $$\begin{array}{c} X=G_{X}^{-1}\left(\int_{-\infty }^{x^{\prime }}f_{X^{\prime }}\left(t\right)dt\right) \end{array}$$

In Equation (1), $\int_{-\infty }^{x}f_{X}\left(t\right)dt$ maps the original data X to the uniform distribution interval [0, 1], representing the cumulative probability value of X in its own distribution. $G_{X^{\prime }}^{-1}$ is the quantile function of the target distribution (uniform or standard normal distribution), which further maps the uniform probability values to the corresponding quantiles of the target distribution and obtains the preprocessed data $X^{\prime }$ ^[30]^. By applying quantile transformation to the sampled reaction conditions, extreme values (outliers) in the original distribution are compressed towards the boundaries of the target distribution, thereby handling skewed data and unifying feature scales. In this work, the uniform distribution is selected for the target distribution. The mapping boundaries for the quantile transformation are determined by the sampling range of the reaction conditions, which are generally positive. Specifically, the upper boundary is extended to 110% of the maximum sampled value, while the lower boundary is set at 90% of the minimum sampled value. Additionally, the preprocessed data $X^{\prime }$ can be transformed back to the original data X through Equation (2).

### 2.2 KAN model

After data preparation, the preprocessed reaction conditions are fitted with experimental reaction labels by the KAN model. KAN is a symbolic regression algorithm and renowned for its exceptional ability to approximate complex continuous functions. The theoretical foundation of KAN is derived from the Kolmogorov‐Arnold representation theorem, proposed by Vladimir Arnold and Andrey Kolmogorov.[^31^, ^32^, ^33^] This theorem states that any multivariate continuous function defined on a finite interval is able to be approximated by a combination of a finite number of univariate continuous functions, as expressed in Equation (3):

(3) $$f\left(x\right)=f\left(x_{1},\cdots ,x_{n}\right)=\sum_{q=1}^{2n+1}\Phi_{q}\left(\sum_{p=1}^{n}\varphi_{q,p}\left(x_{p}\right)\right)$$

where $\varphi_{q,p}$ represents the internal functions and $\Phi_{q}$ denotes the external functions.[^34^, ^35^]

In traditional artificial neural network (ANN), each layer of the network typically employs predefined activation functions (e.g., ReLU or Sigmoid), with the form of these activation functions remaining unchanged throughout the network training process.^[36]^ Although this architecture is simple and efficient, the fixed nature of the activation functions often fails to accommodate the diversity of the data when flexible adjustment is needed.

**FIGURE 3:**
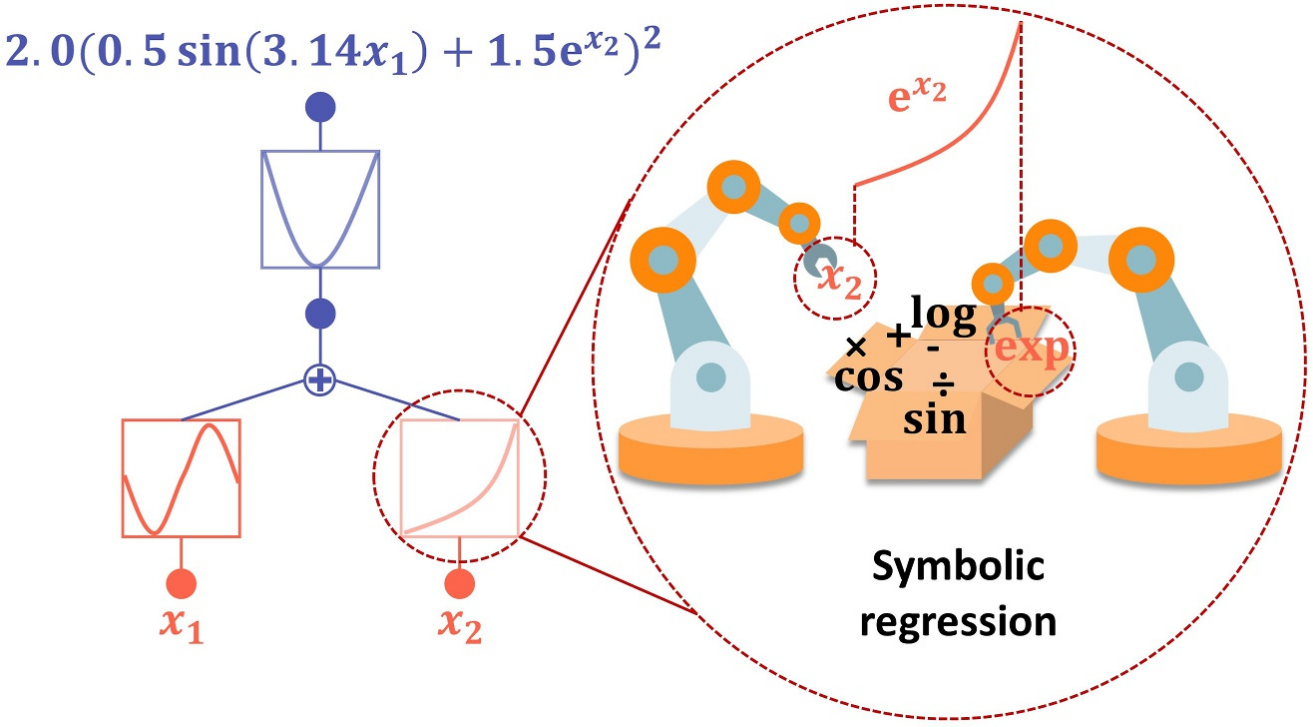
Graphical overview of KAN.

In contrast to traditional ANNs, KAN adopts the principles of the Kolmogorov‐Arnold theorem by replacing fixed activation functions with learnable univariate functions (Figure 3). Rather than scalar weights, each connection employs parameterized univariate functions—typically B‐splines^[35]^—enabling dynamic adjustment of activation functions during training. This architecture grants KAN enhanced flexibility to capture diverse data patterns, achieving modeling of complex nonlinear relationships with fewer parameters than traditional ANNs while providing significantly stronger interpretability.

KAN has three main features:

(1) Excellent nonlinear fitting capability: KAN is capable of approximating any continuous function on a compact subset, provided that there are sufficient learnable components and appropriate weight configurations. Furthermore, the adoption of nonlinear activation functions enhances the fitting capacity of network, enabling the simulation of complex nonlinear relationships within the data.^[37]^
(2) Ideal choice for small sample modeling: The KAN model enhances the capture of localized nonlinear variations within low‐dimensional functions by replacing traditional fixed activation functions with learnable univariate functions. Moreover, the KAN model incorporates a post‐training pruning operation designed to remove redundant function expressions. This pruning yields a more concise network architecture, effectively reducing the risk of overfitting and enhancing generalization performance, particularly under small sample conditions.^[37]^
(3) Strong interpretability: KAN typically consists of explicit layers designed to capture complex relationships among input features. This architecture not only facilitates flexible function modeling but also enhances interpretability, as it enables the derivation of explicit symbolic formulas that relate the target labels to the input features.^[38]^

During model evaluation, performance metrics include the determination coefficient (*R* ^2^) (Equation (4)) and the mean absolute error (MAE) (Equation (5)) for the training set. To prevent overfitting, leave‐one‐out cross‐validation (LOOCV) is employed to assess model generalization ability with *R* ^2^ for the validation set ($Q_{\text{cv}}^{2}$) (Equation (6)).^[30]^

(4) $$\begin{array}{c} R^{2}=1\;-\;\frac{\sum_{i=1}^{n}{\left(y_{i}\;-\;{\hat{y}}_{i}\right)}^{2}}{\sum_{i=1}^{n}{\left(y_{i}\;-\;\bar{y}\right)}^{2}} \end{array}$$

(5) $$\begin{array}{c} \text{MAE}=\frac{1}{n}\sum_{i=1}^{n}\left|y_{i}\;-\;{\hat{y}}_{i}\right| \end{array}$$

(6) $$\begin{array}{c} Q_{\text{cv}}^{2}=1\;-\;\frac{\sum_{i=1}^{n}{\left(y_{i}\;-\;{\hat{y}}_{i}\right)}^{2}}{\sum_{i=1}^{n}{\left(y_{i}\;-\;\bar{y}\right)}^{2}} \end{array}$$

where $y_{i}$ is the experimental value of each sample, ${\hat{y}}_{i}$ is the predicted value corresponding to each sample, and $\bar{y}$ is the average of all experimental values.

The KAN model requires hyperparameter tuning during the training process. The principal hyperparameters of the KAN model and their respective search space are: hidden layer dimension (1≤hid_dim≤10), number of grid intervals (2≤grid≤8), order of piecewise polynomial (1≤k≤5), number of training steps in the original stage (10≤steps_ori≤30), and number of training steps during the pruning stage (5≤steps_prune≤25). In this study, hyperparameter optimization is conducted using the Optuna library,^[39]^ with the objective of maximizing $Q_{\text{cv}}^{2}$, thereby enabling the construction of a KAN model with enhanced robustness.

### 2.3 Bayesian optimization algorithm

Following the fitting of preprocessed reaction conditions using the KAN model, the resulting explicit expression is treated as the target function for subsequent optimization. This study employs the BO algorithm (Nogueira, 2014) to explore reaction conditions to optimize reaction labels. BO is an optimization technique designed for complex functions (e.g., the target function fitted by KAN model) that are computationally expensive, non‐differentiable, or challenging to evaluate directly.^[40]^ By integrating Bayesian statistics with optimization theory, BO efficiently identifies optimal reaction conditions yielding maximum reaction labels.^[41]^ A core component of BO is the construction of a surrogate model,^[42]^ which approximates the target function and guides the search toward unobserved regions of the search space while balancing the trade‐off between exploration of unknown areas and utilization of known promising regions.^[43]^

**FIGURE 4:**
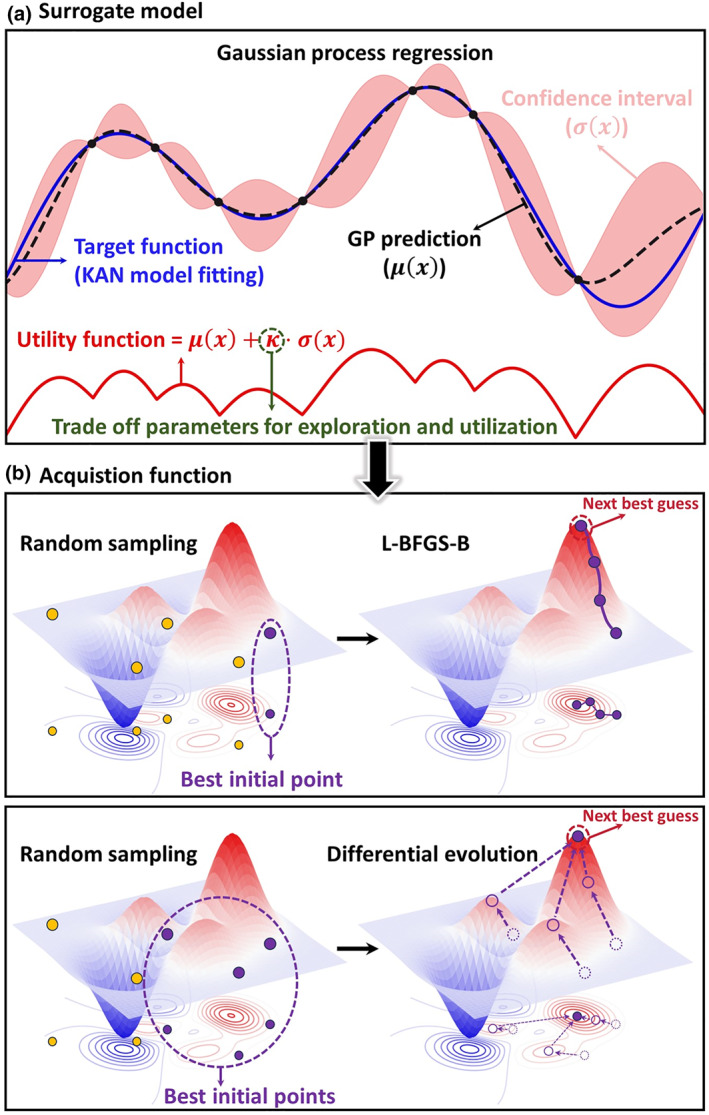
Graphical overview of BO.

Generally, there are two steps to perform a BO technique:

(1) Surrogate model construction (Figure 4a): In this study, GP is adopted as the surrogate model. GP is a probabilistic regression model capable of capturing both the predicted mean and the associated uncertainty over the search space.
(2) Acquisition function optimization (Figure 4b): Acquisition function optimization aims to determine the next evaluation point by maximizing a utility function derived from surrogate model predictions. The procedure involves two key stages. First, an extensive set of reaction conditions is randomly sampled throughout the search space. This initial low‐cost exploration efficiently identifies reaction conditions yielding improved surrogate model predictions. Second, optimizers such as limited‐memory Broyden‐Fletcher‐Goldfarb‐Shanno with bound constraints (L‐BFGS‐B) and differential evolution are employed to find the optimal reaction condition that maximizes the utility function.

The L‐BFGS‐B optimizer is well‐suited for optimization problems involving continuous variables and is employed to refine promising solutions identified through random sampling. Although this process entails relatively high computational costs, it enables more accurate identification of the optimal solution.

The differential evolution optimizer is appropriate for optimization problems involving mixed‐integer variables. This method evolves the population of candidate solutions through mutation, crossover, and selection operations over successive generations, ultimately converging to an optimal solution.

Note that KAN inherently remains a complex and deterministic regression model and it is necessary to introduce GP to approximate KAN. Incorporating a GP provides the essential capability of uncertainty quantification, which enables the BO algorithm to effectively escape local optima and converge to the global optimum of the highly nonlinear KAN model.^[44]^ In addition, KAN does not naturally provide the uncertainty information required for BO. If KAN is used alone as the surrogate model for BO, the optimization process will lack the original BO advantage of the “exploration‐exploitation” balance. However, if the KAN model is modified to incorporate uncertainty quantification capabilities, it can be used alone as a surrogate model, which will be studied as a future work.

Leveraging the potential generalization capability of the KAN model, the search space for decision variables is expanded by 10% beyond the original range (i.e., bounded between 0.9 × minimum and 1.1 × maximum)^[44]^ to explore a wider range of reaction conditions to identify improved reaction performance.

### 2.4 Experimental verification

After BO, the optimal reaction conditions and their corresponding reaction labels are determined. To facilitate experimental validation, the optimized reaction conditions are restored to the original data space using the inverse quantile transformation, as described in Equation (2). This allows for the acquirement of experimentally operable reaction conditions and the conduction of validation experiments. The reaction label values obtained from experiments and the corresponding reaction conditions are added into the original dataset to construct a new training dataset. Subsequently, the updated dataset undergoes a new round of KAN model training, BO, and experimental validation. The optimization loop terminates upon meeting any of the following conditions: reaching the predefined iteration limit, achieving the target performance threshold, or observing no significant improvement over consecutive iterations. In both case studies presented in our work (H_2_O_2_ synthesis and flavonoid synthesis), the optimization is stopped after the target performance threshold is successfully achieved. This closed‐loop strategy, integrating iterative BO refinement with experimental validation, ensures the precise identification of potential reaction conditions yielding satisfactory reaction labels.

### 2.5 BayesianKAN: A website platform for reaction condition optimization

**FIGURE 5:**
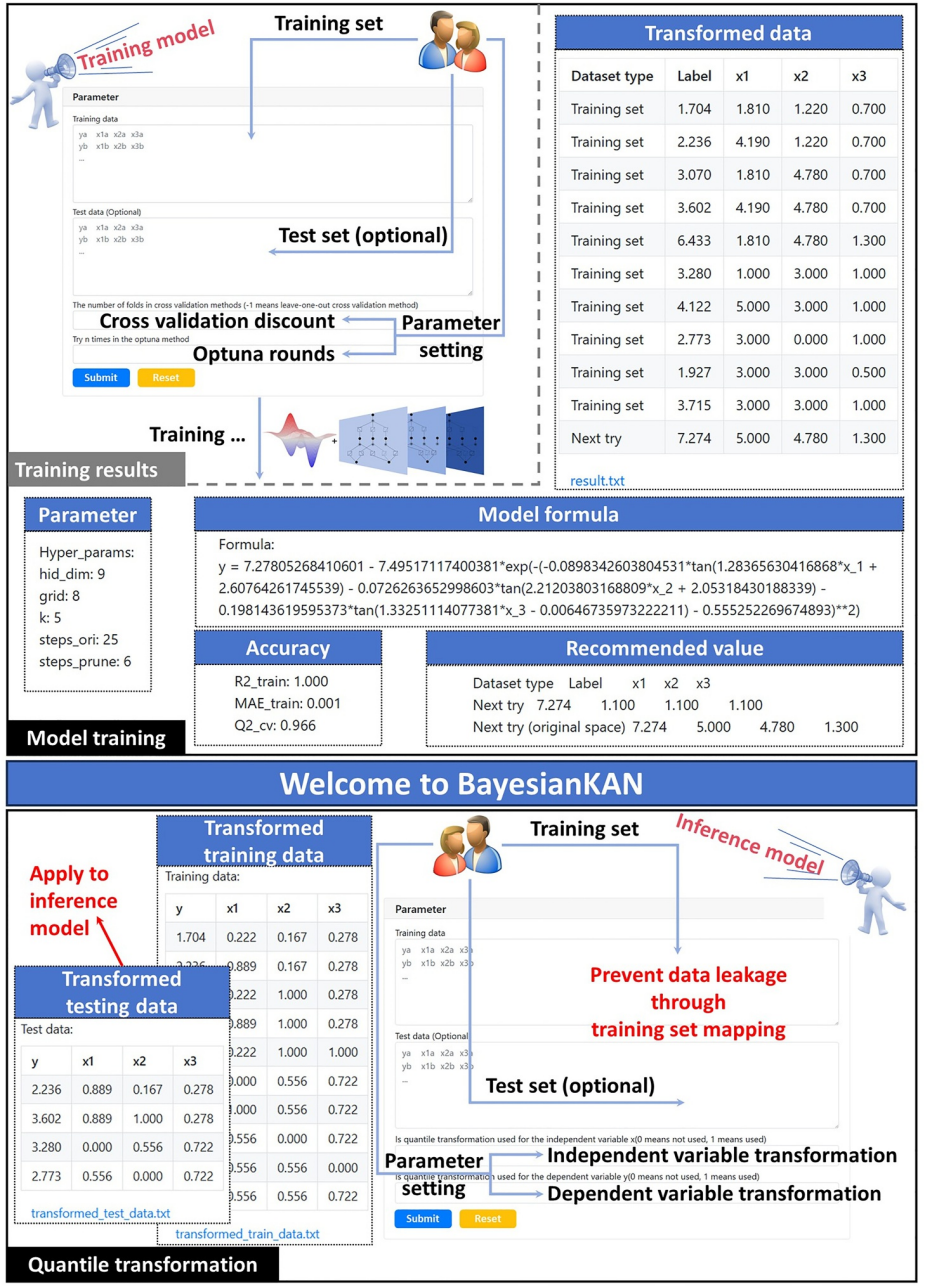
The website platform of BayesianKAN.

A website platform (Figure 5) is developed for the BayesianKAN framework using the PyWebIO library. This platform provides an intuitive and user‐friendly interface to facilitate streamlined implementation of the algorithms and techniques used in this study. Users can access various functional modules, upload datasets, and execute necessary operations. Key functional modules include:

(1) Model training module: The training dataset is uploaded by users, and the hyperparameter search space for the KAN model is defined. Hyperparameters are optimized using the Optuna library. On the basis of the established KAN model, BO is employed to identify the optimal reaction conditions that maximize the reaction label.
(2) Quantile transformation module: The training and testing datasets are uploaded by users, and quantile transformation is subsequently applied using the transformation rules derived from the training set in conjunction with identical parameters specified in the model training module. This approach effectively prevents data leakage while generating transformed results for the testing set, thereby facilitating the subsequent application of the KAN model for inference.

## 3 RESULTS

This section demonstrates the application efficacy of the BayesianKAN platform through two representative case studies, which include a comparative analysis of the KAN model against eight methods: RSM and seven common machine learning approaches (Bayesian ridge (BR), Gaussian process (GP) regression, principal component regression (PCR), partial least squares regression (PLSR), random forest (RF), support vector machine (SVM), and XGBoost), all using the same dataset for consistent evaluation. Hyperparameter search spaces of seven machine learning models are shown in Supporting Information S1: Table S1 in Appendix.

### 3.1 Application of BayesianKAN to H_2_O_2_ synthesis

**FIGURE 6:**
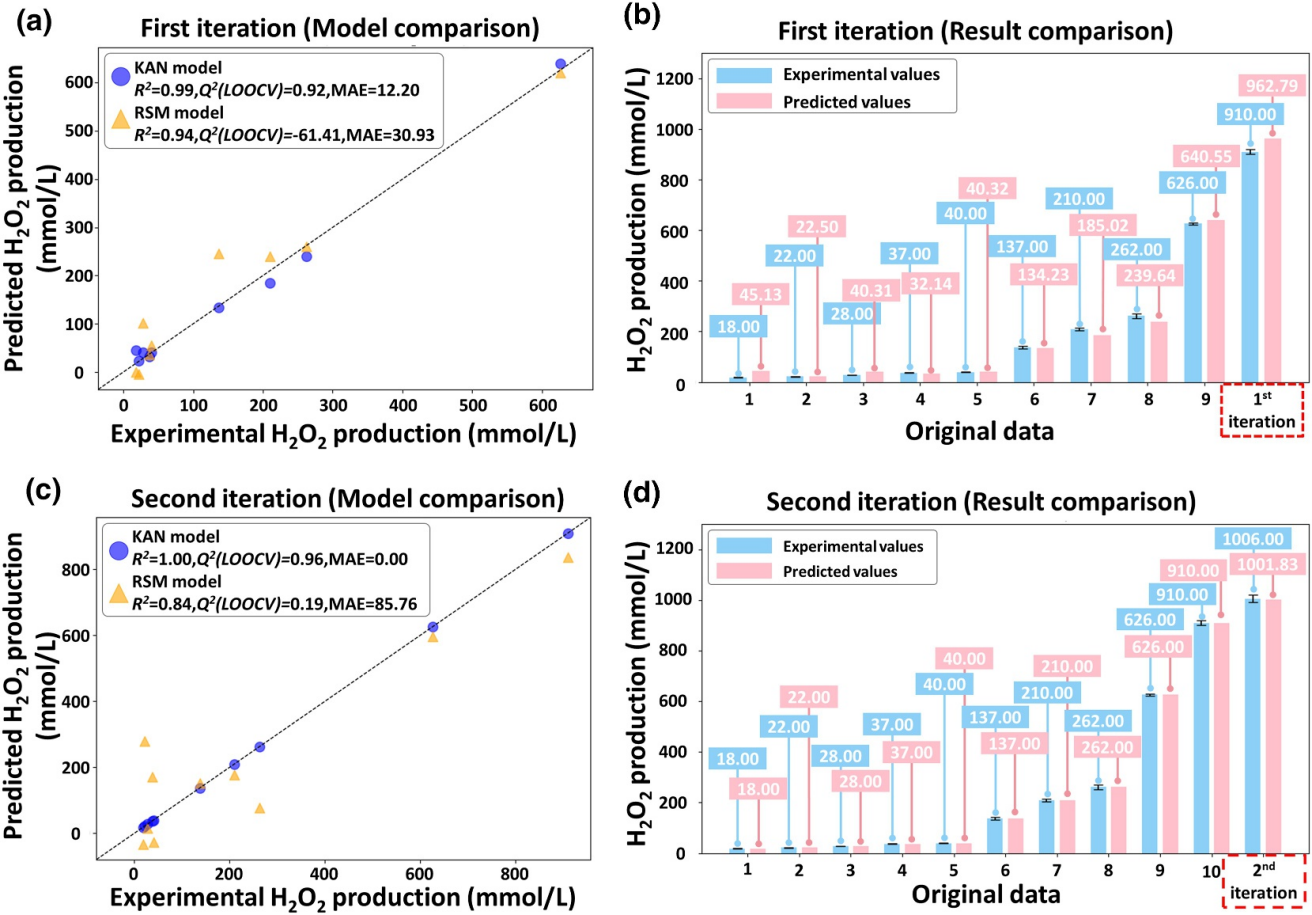
Application of BayesianKAN to H_2_O_2_ production optimization.

As an environmentally friendly and efficient green oxidant, H_2_O_2_ finds widespread application in organic synthesis, wastewater treatment, and energy storage. This case study focuses on the reaction condition optimization for the green synthesis of H_2_O_2_ in photocatalytic systems. The catalyst used, CN‐O‐AQ (core catalyst), features g‐C_3_N_4_ covalently bonded to anthraquinone (AQ) via ester C‐O‐C=O oxygen bridges, forming a silk‐like, ordered stacked layer structure.^[45]^ Under visible‐light irradiation, it exhibits exceptional electron transfer capabilities and efficient photogenerated charge carrier separation, leading to a significant enhancement in H_2_O_2_ production.^[46]^ The original experimental data are obtained using the OFAT method, yielding nine raw H_2_O_2_ production data points corresponding to varied reaction time (h), catalyst concentration (g/mL), proton addition ratio, and reaction temperature (°C) (Table 1). To enhance data robustness and reduce outlier influence, quantile transformation preprocessing is applied to the reaction conditions (see Supporting Information S1: Tables S2 and S3 in Appendix). Note that each experiment is independently repeated three times, and error bars are displayed in Figure 6. The reaction conditions recommended by BayesianKAN in the two rounds of iteration are shown in Table 2 (the values after quantile transformation are provided in Supporting Information S1: Table S4 in Appendix).

**TABLE 1:** The original data, recommended values of BayesianKAN, and experimental validation values in H_2_O_2_ synthesis.

	H_2_O_2_ production (mmol/L)	Reaction time (h)	Catalyst concentration (g/mL)	Proton addition ratio	Reaction temperature (°C)
Original data	18	8	10	0	25
	626	8	10	0.50	25
	262	9	5	0.34	25
	210	16	8.6	0.88	35
	137	7	13	0.93	45
	40	5	4.1	0.14	45
	37	10	10	0.77	40
	28	12	7	0.61	35
	22	14	3.6	0.21	25
1st iteration					
Prediction	962.8	8.6	9.2	0.86	25
Experiment	910	9	9	0.90	25
2nd iteration					
Prediction	1001.8	9	10	0.93	25
Experiment	1006	9	10	1	25

**TABLE 2:** Model accuracy of seven machine learning methods in H_2_O_2_ synthesis.

	Model	BR	GP	PCR	PLSR	RF	SVM	XGB
1st iteration	*R* ^2^	0	0.99	0.68	0.35	0.02	0.31	0.98
	$Q_{\text{cv}}^{2}$	−0.27	−0.36	−4.27	−1.00	−0.25	−0.77	−1.14
	MAE (mmol/L)	141.78	11.85	97.66	127.11	139.24	75.94	25.72
2nd iteration	*R* ^2^	0	1.00	0.80	0.54	0	0.98	0.35
	$Q_{\text{cv}}^{2}$	−0.23	−0.11	−0.86	−0.14	−0.24	0.41	−0.16
	MAE (mmol/L)	222.11	0	118.60	116.44	217.90	12.26	176.57

In the first iteration (Figure 6a), the KAN model demonstrates superior performance with *R* ^2^ = 0.99, $Q_{\text{cv}}^{2}=0.92$, and MAE = 12.20 mmol/L. In contrast, RSM yields *R* ^2^ = 0.94, $Q_{\text{cv}}^{2}=-61.41$, and MAE = 30.93 mmol/L. The accuracy of seven machine learning methods is shown in Table 2. This indicates the substantial advantages of KAN in predictive accuracy, error control, and generalization capability. BayesianKAN predicts an H_2_O_2_ production of 962.8 mmol/L (Table 1), showing a 5.80% relative error versus the experimental value (910 mmol/L). This optimized value represents a 45.37% increase over the maximum H_2_O_2_ production in the original dataset (Figure 6b).

In the second iteration (Figure 6c), the KAN model achieves *R* ^2^, $Q_{\text{cv}}^{2}$, and MAE values of 1.00, 0.96, and 0 mmol/L respectively, significantly surpassing RSM (*R* ^2^ = 0.84, $Q_{\text{cv}}^{2}=0.19$, and MAE = 85.76 mmol/L). The accuracy of seven machine learning methods is shown in Table 2. BayesianKAN yields a final H_2_O_2_ production of 1001.8 mmol/L (Table 1), exhibiting merely 0.4% relative error versus the experimental value (1006 mmol/L). This represents a 10.5% increase over the recommendation in first iteration (Figure 6d). After two iterations, the final H_2_O_2_ production increases by 60.7%, demonstrating the efficacy of BayesianKAN in optimizing reaction conditions and enhancing H_2_O_2_ production. The success of this optimization demonstrates that BayesianKAN is robust when applied to the non‐uniform OFAT sampling strategy.

According to the $Q_{\text{cv}}^{2}$ comparison between KAN and GP in Table 2, KAN demonstrates a stronger ability to correlate reaction conditions with H_2_O_2_ productions than GP. This indicates that, compared to typical GP‐based BO methods, BayesianKAN is able to more precisely predict H_2_O_2_ productions in each BO iteration and thus optimize reaction conditions for H_2_O_2_ synthesis more efficiently.

Furthermore, the KAN model demonstrates high interpretability through explicit formulas. With the aim of clearly articulating the nonlinear relationships between key input variables (reaction time, catalyst concentration, proton addition ratio, reaction temperature) and H_2_O_2_ production,^[47]^ the coefficients are simplified by retaining three decimal places. The resulting formula from the second‐iteration is presented in Equations ((7), (8), (9), (10), (11), (12)), while the first‐iteration and unsimplified second‐iteration formulas are presented in Supporting Information S1: Equations (A1–A12) in Appendix, respectively.

(7) $$y=123.055{\left({y_{3}\;-\;y}_{1}\;-\;y_{2}+0.168+y_{4}\right)}^{2}+y_{5}\;-\;42.972$$

(8) $$\begin{array}{c} y_{1}=0.889\;\sin\left(5.314x_{4}^{\prime }+3.629\right) \end{array}$$

(9) $$\begin{array}{c} y_{2}=0.073\;\tan\left(1.511x_{1}^{\prime }+8.428\right) \end{array}$$

(10) $$\begin{array}{c} y_{3}=\mathrm{atan}\left(4.697x_{3}^{\prime }\;-\;1.484\right) \end{array}$$

(11) $$\begin{array}{c} y_{4}=0.685e^{-6.744{\left(0.675-x_{2}^{\prime }\right)}^{2}} \end{array}$$

(12) $$y_{5}=\;42.076\;\sin\left(-13.332{\left(0.466-x_{1}^{\prime }\right)}^{2}+2.124\;\cos\left(6.117x_{3}^{\prime }+4.134\right)+5.453\right)$$

where $x_{1}^{\prime },x_{2}^{\prime }{,x}_{3}^{\prime }{,x}_{4}^{\prime }$ correspond to the preprocessed reaction conditions (reaction time, catalyst concentration, proton addition ratio, and reaction temperature) after quantile transformation, and y represents the H_2_O_2_ production.

The derived KAN formulas offer interpretability for the reaction mechanisms underlying H_2_O_2_ synthesis. Note that the interpretability offered by KAN refers specifically to mathematical transparency,^[48]^ achieved via symbolic regression to derive explicit functional expressions, rather than from first‐principles physical derivation. Although these functions are empirical approximations, they often mirror the mathematical structure of classical physicochemical models, offering a transparent mapping of reaction mechanisms. For instance, Equation (10) employs an arctangent function to describe the effect of the proton addition ratio ($x_{3}^{\prime }$). As this ratio increases, the calculated value rises sharply before plateauing—a mathematical behavior that mirrors the experimentally observed “saturation effect” arising from kinetic bottlenecks. Notably, the arctangent function is frequently used to approximate the Langmuir adsorption isotherm, which describes the saturation limit of molecular adsorption on surfaces. This alignment demonstrates that the KAN model has autonomously captured the underlying physical mechanism governing proton addition, thereby validating the physical interpretability of the derived equations.

**FIGURE 7:**
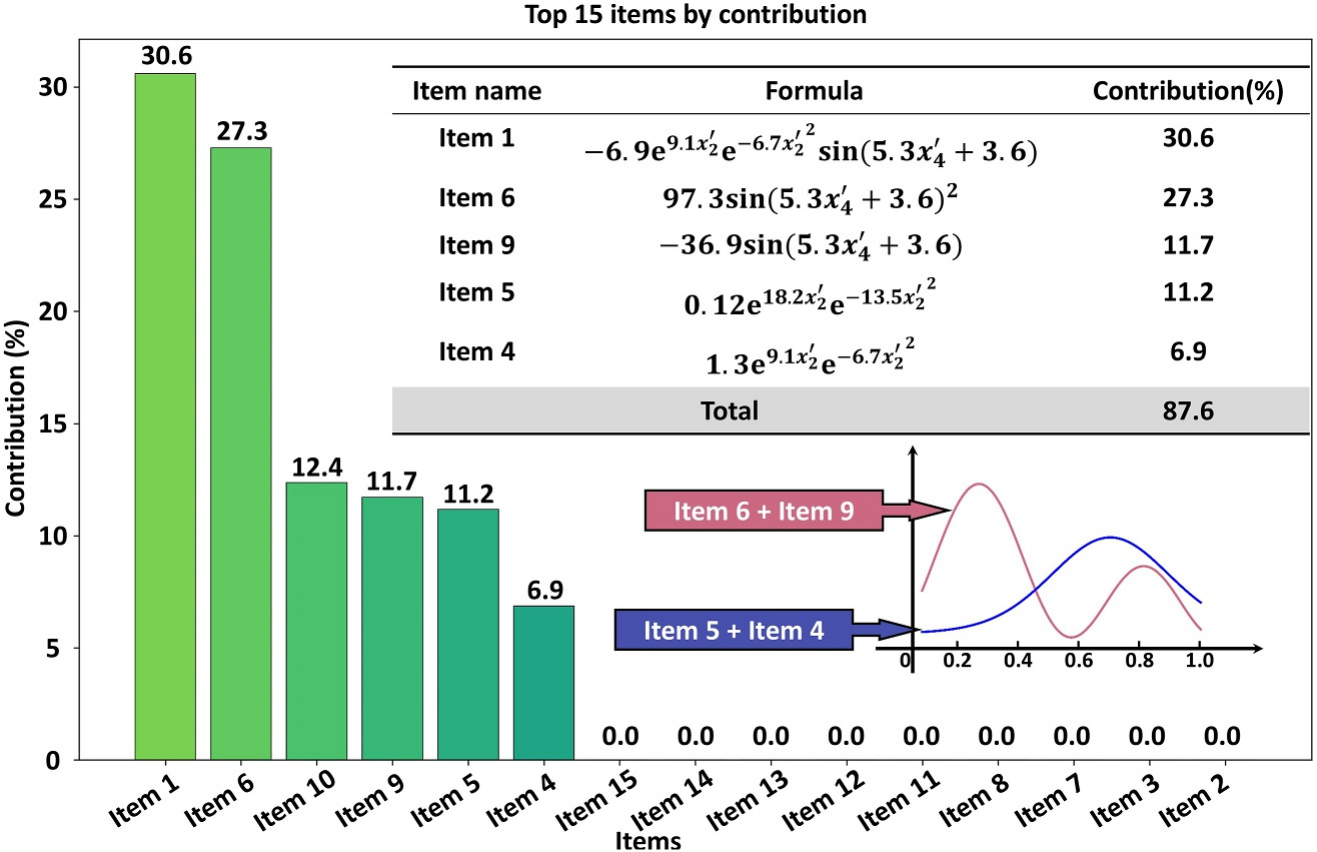
The results of the dominant item analysis for the second iteration formula in H_2_O_2_ synthesis.

The presence of a squared term on the right‐hand side impedes direct dominant term analysis, consequently, the equation is fully expanded (see Supporting Information S1: Equation (A13) in Appendix for details). A dominant item analysis is then performed on this expanded equation, the results of which are illustrated in Figure 7, while the identified dominant terms and their corresponding formulas are also detailed in Figure 7. As indicated by the table in Figure 7, the production of H_2_O_2_ is primarily governed by $x_{2}^{\prime }{,x}_{4}^{\prime }$. Items 5 and 4 are integrated into an independent equation representing $x_{2}^{\prime }$, and the blue curve in Figure 7 is plotted based on the quantile domain of [0, 1]. Analysis of this curve reveals that elevated catalyst concentrations ($x_{2}^{\prime }$) are conducive to H_2_O_2_ production. This enhancement is attributed to the direct augmentation of the total number of active sites within the reaction system. However, excessive catalyst concentrations ($x_{2}^{\prime }$) may induce solution turbidity, thereby precipitating light shielding or scattering effects. Similarly, items 6 and 9 are visualized as the red curve in Figure 7 over the same domain, indicating that lower reaction temperature ($x_{4}^{\prime }$) favor H_2_O_2_ production. Given the minimum training reaction temperature ($x_{4}^{\prime }$) of 25°C, this trend is primarily ascribed to the suppression of the thermal decomposition of the product at lower temperatures.

### 3.2 Application of BayesianKAN to flavone synthesis

Flavone is an important natural product, exhibiting significant pharmacological activities such as antioxidant, anticancer, and anti‐inflammatory effects. This case study focuses on the reaction condition optimization for the green synthesis of flavone in photocatalytic systems. The catalyst used, cationic Eosin Y, demonstrates excellent photogenerated electron transfer capabilities and hydrogen release efficiency under blue light irradiation (440 nm), thereby enabling the efficient dehydrogenation of flavanones to produce flavones under metal‐free conditions without hydrogen atom transfer reagents.[^49^, ^50^] The original experimental data are obtained through the CCD in the DoE method, sampling 10 raw flavone yield data points corresponding to varied Eosin Y concentration (mol%), HClO_4_ concentration (equiv), and reaction time (h) (Table 3). To enhance data robustness and reduce outlier influence, quantile transformation preprocessing is applied to the reaction conditions (see Supporting Information S1: Tables S5–S7 in Appendix). In this case study, each reaction condition is tested in a single‐run experiment without replication. This is because the experiments are conducted on a 0.1 mmol micro‐scale, which exhibits low systematic errors, and the overall trend within the DoE parameter space remains robust against minor random fluctuations in individual data points.^[51]^ The reaction conditions recommended by BayesianKAN in the three rounds of iteration are shown in Table 3 (the values after quantile transformation are provided in Supporting Information S1: Table S8 in Appendix).

**TABLE 3:** The original data, recommended values of BayesianKAN, and experimental validation values in flavone synthesis.

	Yield	Eosin Y concentration (mol%)	HClO_4_ concentration (equiv)	Reaction time (h)
Original data	0.097	1.81	1.22	0.70
	0.77	4.19	1.22	0.70
	0.132	1.81	4.78	0.70
	0.779	4.19	4.78	0.70
	0.897	1.81	4.78	1.30
	0.855	1.00	3.00	1.00
	0.894	5.00	3.00	1.00
	0.241	3.00	0	1.00
	0.615	3.00	3.00	0.50
	0.884	3.00	3.00	1.00
	0.903	3.45	1.56	1.83
	0.911	2.79	1.91	1.65
1st iteration				
Prediction	0.952	3.79	1.26	1.14
Experiment	0.926	4.19	1.22	1.30
2nd iteration				
Prediction	0.939	1.81	1.22	1.30
Experiment	0.940	1.81	1.22	1.30
3rd iteration				
Prediction	0.960	3.26	4.78	1.81
Experiment	0.958	3.40	5.00	2.00

**FIGURE 8:**
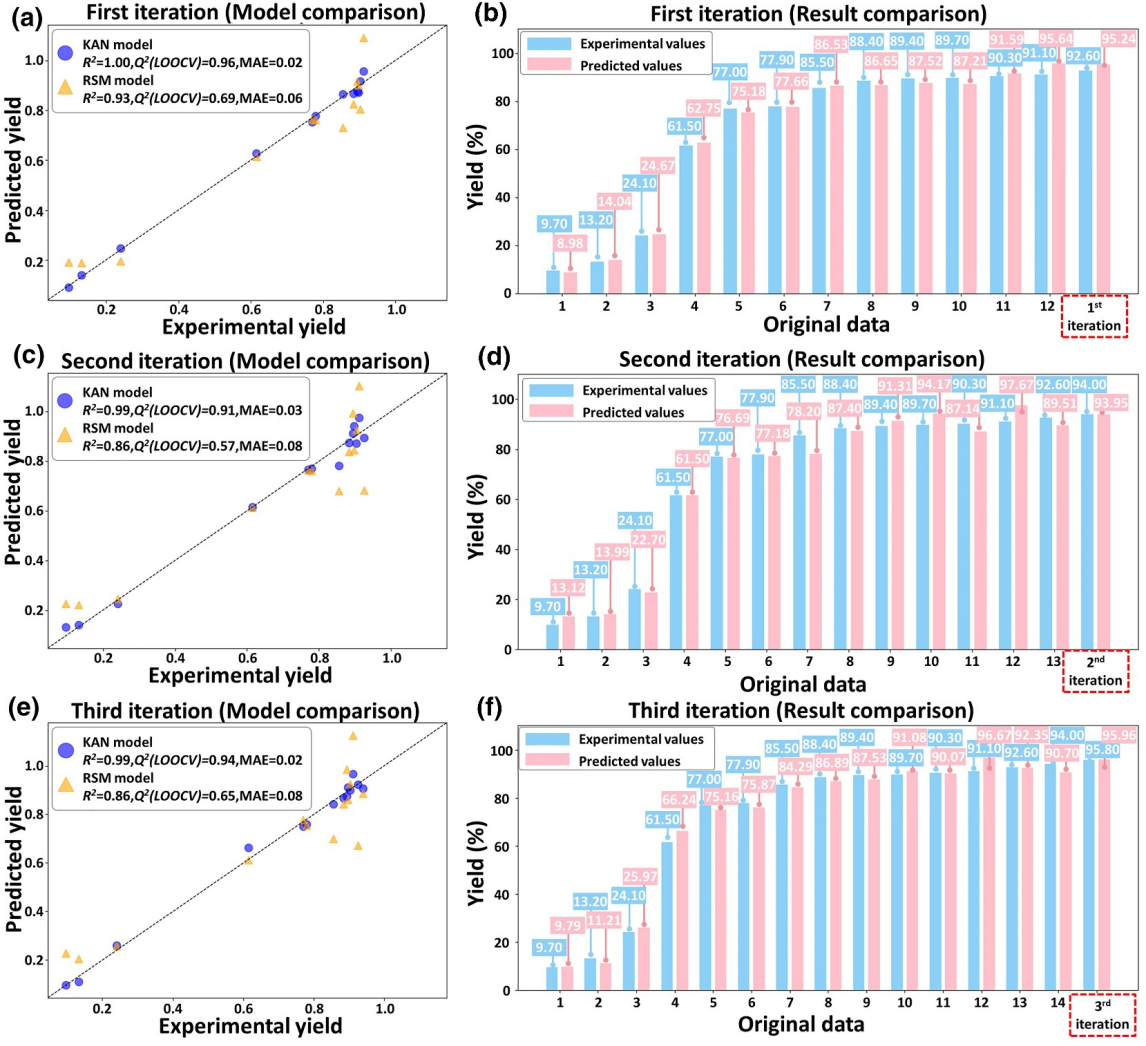
Application of BayesianKAN to flavone yield optimization.

In the first iteration (Figure 8a), the KAN model demonstrates superior performance with *R* ^2^ = 1.00, $Q_{\text{cv}}^{2}=0.96$, and MAE = 0.02, while RSM yields *R* ^2^ = 0.93, $Q_{\text{cv}}^{2}=0.69$, and MAE = 0.06. The accuracy of seven machine learning methods is shown in Table 4. These results indicate that the KAN model not only maintains high fitting accuracy but also demonstrates high robustness in terms of its $Q_{\text{cv}}^{2}$, with no risk of overfitting. BayesianKAN predicts a flavone yield of 95.2% (Table 3), with only a 2.81% relative error from the experimental value of 92.6%. This optimized value represents a 1.6% improvement over the maximum flavone yield in the original dataset (Figure 8b).

**TABLE 4:** Model accuracy of seven machine learning methods in flavone synthesis.

	Model	BR	GP	PCR	PLSR	RF	SVM	XGB
1st iteration	*R* ^2^	0	1.00	0.56	0.56	0	0.63	−0.01
	$Q_{\text{cv}}^{2}$	−0.20	0.05	0.07	0.07	−0.23	0.37	−0.11
	MAE	0.25	0	0.16	0.16	0.26	0.18	0.25
2nd iteration	*R* ^2^	0.60	1.00	0.60	0.60	0.90	0.87	−0.01
	$Q_{\text{cv}}^{2}$	0.18	0.22	0.17	0.17	0.09	0	−0.09
	MAE	0.15	0	0.15	0.15	0.07	0.07	0.24
3rd iteration	*R* ^2^	0.54	0.98	0.55	0.55	0.89	0.96	−0.01
	$Q_{\text{cv}}^{2}$	0.10	0.10	0.10	0.10	0.08	0.06	−0.07
	MAE	0.16	0.03	0.16	0.16	0.07	0.05	0.24

In the second iteration (Figure 8c), the KAN model achieves *R* ^2^, $Q_{\text{cv}}^{2}$, and MAE values of 0.99, 0.91, and 0.03, respectively, while RSM yields *R* ^2^, $Q_{\text{cv}}^{2}$, and MAE values of 0.86, 0.57, and 0.08. The accuracy of seven machine learning methods is shown in Table 4. BayesianKAN predicts a flavone yield of 93.9% (Table 3), with a 0.11% relative error from the experimental value of 94.0%. This represents a 1.51% increase over the recommendation in first iteration (Figure 8d).

In the third iteration (Figure 8e), the KAN model achieves *R* ^2^, $Q_{\text{cv}}^{2}$, and MAE values of 0.99, 0.94, and 0.02, respectively, while RSM yields *R* ^2^, $Q_{\text{cv}}^{2}$, and MAE values of 0.86, 0.65, and 0.08. The accuracy of seven machine learning methods is shown in Table 4. BayesianKAN predicts a flavone yield of 96.0% (Table 3), with a 0.21% relative error from the experimental value of 95.8%. The recommended value in the third iteration represents a 1.91% improvement over the second iteration (Figure 8f). After three iterations, the final flavone yield increases by 5.2%, demonstrating the efficacy of BayesianKAN in optimizing reaction conditions and enhancing flavone yield.

According to the $Q_{\text{cv}}^{2}$ comparison between KAN and GP in Table 4, KAN demonstrates a stronger ability to correlate reaction conditions with flavone yields than GP. This indicates that, compared to typical GP‐based BO methods, BayesianKAN is able to more precisely predict flavone yields in each BO iteration and thus optimize reaction conditions for flavone synthesis more efficiently.

Furthermore, the KAN model demonstrates high interpretability through explicit formulas. With the aim of clearly articulating the nonlinear relationships between key input variables (Eosin Y concentration, HClO_4_ concentration, reaction time) and flavone yield, the coefficients are simplified to three decimal places. The equations resulting from the third iteration are presented in Equations ((13), (14), (15), (16)), while the first‐iteration, second‐iteration, and unsimplified third‐iteration formulas are presented in Supporting Information S1: Equations (A14–A25) in Appendix, respectively. Equations ((13), (14), (15), (16)) represent the third‐iteration formula, quantifying nonlinear relationships between key input variables (Eosin Y concentration, HClO_4_ concentration, reaction time) and flavone yield.

(13) $$\begin{array}{c} y=0.970\;-\;5.223{\left(y_{1}+y_{2}\;-\;y_{3}+0.143\right)}^{2} \end{array}$$

(14) $$\begin{array}{c} y_{1}=-0.748{\left(0.276\;-\;x_{1}^{\prime }\right)}^{2} \end{array}$$

(15) $$\begin{array}{c} y_{2}={\left(0.553\;-\;x_{2}^{\prime }\right)}^{2} \end{array}$$

(16) $$\begin{array}{c} y_{3}=0.168\;\cos\left(4.351x_{3}^{\prime }+8.584\right) \end{array}$$

where $x_{1}^{\prime }{,x}_{2}^{\prime },x_{3}^{\prime }$ correspond to the preprocessed reaction conditions (Eosin Y concentration, HClO_4_ concentration, and reaction time) after quantile transformation, and y represents the flavone yield.

The derived KAN formulas provide interpretability for the reaction mechanisms in flavone synthesis. For example, Equation (14) employs a quadratic function to describe the effect of the Eosin Y concentration ($x_{1}^{\prime }$). This formulation reveals a non‐linear optimum, where both excessive and insufficient catalyst loadings lead to reduced flavone yields. This behavior aligns with the Sabatier principle, which states that optimal catalytic performance is achieved only when the interaction between the catalyst and reactive intermediates is appropriately balanced. The emergence of this nonlinear optimum suggests that the KAN model has captured chemically meaningful relationships within the reaction system, thereby supporting the physical interpretability of the derived symbolic equations.

**FIGURE 9:**
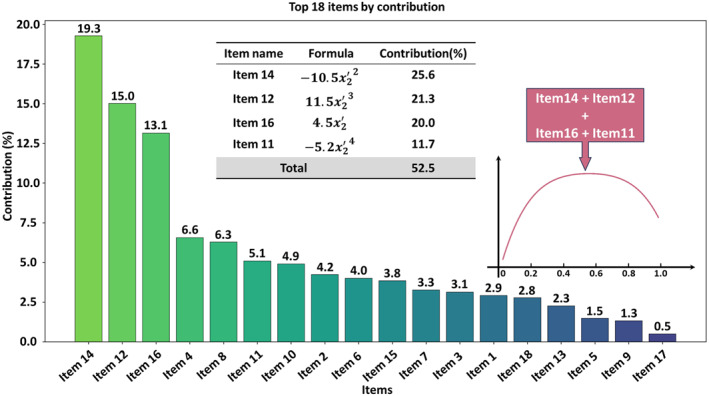
The results of the dominant item analysis for the third iteration formula in flavone synthesis.

The presence of a squared term on the right‐hand side impedes direct dominant term analysis, consequently, the equation is fully expanded (see Supporting Information S1: Equation (A26) in Appendix for details). Dominant item analysis is subsequently performed on the expanded formulation, with the results depicted in Figure 9. By correlating the top‐ranking terms with their respective formulas, it is observed that the first eight terms, which constitute 52.5% of the contribution, are exclusively dependent on $x_{2}^{\prime }$. This identifies $x_{2}^{\prime }$ as the governing factor. These items are integrated into a unified formula, and the corresponding curve is plotted over the quantile‐transformed domain [0, 1] (red curve in Figure 9). The overall trajectory exhibits an initial increase, followed by a plateau, and finally a decline. This indicates that while the flavone synthesis yield remains constant within an appropriate HClO_4_ concentration range, deviations from this window result in drastic variations. Such behavior suggests that HClO_4_ concentration functions as a “regulatory switch”. Moderate acidity is requisite to protonate Eosin Y into a highly active cationic form, thereby initiating the Hydrogen atom transfer process and assisting the hydrogen evolution cycle. However, excessive acidity is shown to thermodynamically inhibit flavone formation and may compromise the stability of the catalytic system, confirming the existence of an optimal concentration window.

## 4 CONCLUSIONS

Optimizing reaction conditions is critical for efficient chemical synthesis. This work proposes a novel reaction condition optimization framework, BayesianKAN, by integrating BO algorithm with KAN model. Two case studies are presented to highlight the feasibility and effectiveness of the proposed framework. It achieves a dramatic 60.7% improvement in H_2_O_2_ production (from 626 to 1006 mmol/L) after merely two iterations. Similarly, it boosts flavone yield by 5.2% (from 91.1% to 95.8%) after just three iterations. Compared to RSM and other seven common machine learning approaches, KAN demonstrates superior fitting accuracy and generalization ability, with *R* ^2^ values approaching 1.0 and $Q_{\text{cv}}^{2}$ values exceeding 0.9. Additionally, unlike machine learning methods (e.g., GP), the inherent mathematical transparency of KAN directly reveals the nonlinear relationship between reaction conditions and outcomes (e.g., product yield) through explicit formulas. However, the derived formulations are still complex to some extent, as the a priori unknown reaction mechanisms necessitate a data‐driven approach employing full basis functions for expression fitting. In future cases with well‐understood reaction mechanisms, incorporating prior domain knowledge as physical constraints during training could further enhance the conciseness and interpretability of the resulting KAN formulations. Concurrently, future work will expand BayesianKAN to navigate the molecular design space, enabling the efficient discovery of discrete molecular structures with desired properties using minor experimental data and key molecular descriptors. This powerful approach holds immense promise for drastically accelerating materials discovery cycles and propelling the development of next‐generation, sustainable high‐performance materials in chemical engineering and materials science.

## AUTHOR CONTRIBUTIONS

**Juntao Wang**: Conceptualization; methodology; software; formal analysis; data curation; writing—original draft; visualization. **Yujing Zhao**: Conceptualization; methodology; writing—review and editing; supervision; project administration. **Peiyu Yi**: Validation; investigation; resources. **Liyuan Duan**: Validation; investigation; resources. **Qingwei Meng**: Supervision; project administration; funding acquisition. **Qilei Liu**: Conceptualization; methodology; software; formal analysis; writing—review and editing; supervision; project administration; funding acquisition.

## CONFLICT OF INTEREST STATEMENT

The authors declare no conflict of interest.

## ETHICS STATEMENT

No animal or human experiments were involved in this study.

## Supporting information

Supporting Information S1

## Data Availability

The KAN models developed in this work are presented in Equations ((7), (8), (9), (10), (11), (12), (13), (14), (15), (16)). The codes for training KAN are available on GitHub (https://github.com/KindXiaoming/pykan). The datasets used for training KAN can be found in Tables 1 and 3.
