# Supplementary material for: BayesianKAN: A reaction condition optimization framework integrating Kolmogorov‐Arnold network and Bayesian optimization
Source: Smart Mol. 2026 Aug 5:e70082. Online ahead of print. doi: 10.1002/smo2.70082 (PMC13443980; doi:10.1002/smo2.70082)
Supplement: Supplementary file 1 [file SMO2-9999-0-s001.docx]

**BayesianKAN: A Reaction Condition Optimization Framework Integrating Kolmogorov-Arnold Network and Bayesian Optimization**

Juntao Wang^a^, Yujing Zhao^a,b*^, Peiyu Yi^a^, Liyuan Duan^a^, Qingwei Meng^a,c^, Qilei Liu^a,c*^

^a^State Key Laboratory of Fine Chemicals, Department of Pharmaceutical Sciences, Institute of Chemical Process Systems Engineering, School of Chemical Engineering, Dalian University of Technology, Dalian 116024, China

^b^MOE Key Laboratory of Bio-Intelligent Manufacturing, School of Bioengineering, Dalian University of Technology, Dalian 116024, China

^c^Ningbo Institute of Dalian University of Technology, Ningbo 315016, China

**Corresponding author(s): Yujing Zhao (zhaoyujing@dlut.edu.cn), Qilei Liu (liuqilei@dlut.edu.cn)*

**Supporting Information**

**Contents**

[**1 Hyperparameter search space for model comparison**	3](#_Toc232437204)

[**2 Application of BayesianKAN to H_2_O_2_ synthesis**	5](#_Toc232437205)

[**2.1 Quantile transformed data**	5](#_Toc232437206)

[**2.2 Explicit formulas**	6](#_Toc232437207)

[**2.3 Expanded formula**	9](#_Toc232437208)

[**3 Application of BayesianKAN to flavone synthesis**	10](#_Toc232437209)

[**3.1 Quantile transformed data**	10](#_Toc232437210)

[**3.2 Explicit formulas**	12](#_Toc232437211)

[**3.3 Expanded formula**	15](#_Toc232437212)

# **1 Hyperparameter search space for model comparison**

**Tab. S1** Hyperparameter search spaces of seven machine learning models.

| **Model** | **Hyperparameter** | **Type** | **Min** | **Max** |
| --- | --- | --- | --- | --- |
| br | alpha_1 | float | 1e-6 | 1e-1 |
|  | alpha_2 | float | 1e-6 | 1e-1 |
|  | lambda_1 | float | 1e-6 | 1e-1 |
|  | lambda_2 | float | 1e-6 | 1e-1 |
| gpr | kernel_type | categorical | 'rbf', 'dotproduct', 'matern', 'rationalquadratic', 'rbf+dotproduct' | |
|  | alpha | float | 1e-12 | 1e-1 |
|  | length_scale | float | 1e-2 | 1e2 |
|  | n_restarts_optimizer | int | 0 | 10 |
| pcr | n_components \| int \| 1 \| min(x_train.shape[0], x_train.shape[1]) | | | |
| plsr | n_components | int | 1 | min(x_train.shape[0], x_train.shape[1]) |
| rf | n_estimators | int | 10 | 500 |
|  | max_depth | int | 3 | 10 |
|  | min_samples_split | int | 2 | 20 |
|  | min_samples_leaf | int | 1 | 10 |
|  | max_features | categorical | 'sqrt', 'log2', None | |
| svm | kernel | categorical | 'linear', 'poly', 'rbf', 'sigmoid' | |
|  | C | float | 1e-3 | 1e3 |
|  | epsilon | float | 1e-3 | 1 |
|  | gamma | categorical | 'scale', 'auto' | |
| xgb | max_depth | int | 3 | 10 |
|  | learning_rate | float | 0.01 | 0.3 |
|  | n_estimators | int | 5 | 500 |
|  | min_child_weight | int | 1 | 10 |
|  | subsample | float | 0.5 | 1 |
|  | colsample_bytree | float | 0.5 | 1 |
|  | gamma | float | 0 | 10 |
|  | reg_alpha | float | 1e-8 | 1 |
|  | reg_lambda | float | 1e-8 | 10 |

# **2 Application of BayesianKAN to H_2_O_2_ synthesis**

## **2.1 Quantile transformed data**

**Tab. S2** Reaction condition data after quantile transformation in the first iteration

| Reaction time | Catalyst concentration | Proton addition ratio | Reaction temperature |
| --- | --- | --- | --- |
| 0.35 | 0.7 | 0.1 | 0.25 |
| 0.35 | 0.7 | 0.5 | 0.25 |
| 0.50 | 0.3 | 0.4 | 0.25 |
| 0.90 | 0.5 | 0.8 | 0.50 |
| 0.20 | 0.9 | 0.9 | 0.85 |
| 0.10 | 0.2 | 0.2 | 0.85 |
| 0.60 | 0.7 | 0.7 | 0.70 |
| 0.70 | 0.4 | 0.6 | 0.50 |
| 0.80 | 0.1 | 0.3 | 0.25 |

**Tab. S3** Reaction condition data after quantile transformation in the second iteration

| Reaction time | Catalyst concentration | Proton addition ratio | Reaction temperature |
| --- | --- | --- | --- |
| 0.318182 | 0.727273 | 0.090909 | 0.272727 |
| 0.318182 | 0.727273 | 0.454545 | 0.272727 |
| 0.500000 | 0.272727 | 0.363636 | 0.272727 |
| 0.909091 | 0.454545 | 0.727273 | 0.590909 |
| 0.181818 | 0.909091 | 0.909091 | 0.863636 |
| 0.090909 | 0.181818 | 0.181818 | 0.863636 |
| 0.636364 | 0.727273 | 0.636364 | 0.727273 |
| 0.727273 | 0.363636 | 0.545455 | 0.590909 |
| 0.818182 | 0.090909 | 0.272727 | 0.272727 |

**Tab. S4** The value of the independent variable recommended by BayesianKAN in the two rounds of iteration (the value after quantile transformation)

|  | Reaction  time | Catalyst concentration | Proton addition ratio | Reaction temperature |
| --- | --- | --- | --- | --- |
| 1^st^ iteration | 0.5 | 0.528571 | 0.840000 | 0.250000 |
| 2^nd^ iteration | 0.5 | 0.727273 | 0.977517 | 0.272727 |

## **2.2 Explicit formulas**

$$\begin{aligned} y=y_{1}+y_{2}+y_{3}+y_{4}+y_{5}+1121.57851428425\#\left( A1 \right) \end{aligned}$$

$$\begin{aligned} y_{1}=a_{1}\left( a_{2}\sin\left( a_{3}x_{1}^{'}-a_{4} \right)+a_{5}\sin\left( a_{6}x_{4}^{'}+a_{7} \right)+a_{8}\cos\left( a_{9}x_{2}^{'}-a_{10} \right)-1 \right)^{2}\#\left( A2 \right) \end{aligned}$$

$$\begin{aligned} y_{2}=b_{1}\left( \begin{aligned} b_{2}\sin\left( b_{3}x_{2}^{'}-b_{4} \right)-\sin\left( b_{5}x_{4}^{'}+b_{6} \right)+b_{7}\cos\left( b_{8}x_{1}^{'}+b_{9} \right) \\ +b_{10}\cos\left( b_{11}x_{3}^{'}-b_{12} \right)-b_{13} \end{aligned} \right)^{2}\#\left( A3 \right) \end{aligned}$$

$$\begin{aligned} y_{3}=c_{1}e^{c_{2}\sin\left( c_{3}x_{3}^{'}+c_{4}+c_{5}\sin\left( c_{6}x_{4}^{'}-c_{7} \right)-c_{8}\cos\left( c_{9}x_{1}^{'}-c_{10} \right)-c_{11}cos(c_{12}x_{2}^{'}+c_{13}) \right)}\#\left( A4 \right) \end{aligned}$$

$$\begin{aligned} y_{4}=d_{1}sin(d_{2}\sin\left( d_{3}x_{3}^{'}-d_{4} \right)+d_{5}\cos\left( d_{6}x_{4}^{'}-d_{7} \right)-d_{8})\#\left( A5 \right) \end{aligned}$$

$$\begin{aligned} y_{5}=\alpha_{1}\tan\left( \begin{aligned} \alpha_{2}\left( \alpha_{3}x_{1}^{'}+1 \right)^{\frac{2}{3}}+{\alpha_{4}\left( \alpha_{5}x_{2}^{'}-1 \right)}^{5}-\alpha_{6}\sin\left( \alpha_{7}x_{3}^{'}-\alpha_{8} \right) \\ +\alpha_{9}\sin\left( \alpha_{10}x_{4}^{'}+\alpha_{11} \right)+\alpha_{12} \end{aligned} \right)\#\left( A6 \right) \end{aligned}$$

**Eqs. (A1-A6)** represent the explicit formulas of the KAN model in the first iteration, where $x_{1}^{'},x_{2}^{'},x_{3}^{'},x_{4}^{'}$ correspond to the preprocessed reaction conditions (reaction time, catalyst concentration, proton addition ratio, and reaction temperature, respectively) after quantile transformation. $y$ denotes the H_2_O_2_ production.

$$a_{1}=-38.056603414389,a_{2}=-0.140035829641894,a_{3}=2.78354160486481,$$

$$a_{4}=7.99753059915949,a_{5}=0.786777919266576,a_{6}=4.93504159418132,$$

$$a_{7}=3.85050735096342,a_{8}=0.291884515702946,a_{9}=2.99243543020647,$$

$$a_{10}=9.20237789334586,$$

$$b_{1}=186.942804996583,b_{2}=-0.0945508493449139, b_{3}=3.20452430174839,$$

$$b_{4}=1.37651292321202,b_{5}=4.73385863219205, b_{6}=7.44747473862545,$$

$$b_{7}=0.109626669130347,b_{8}=4.69571889941868,b_{9}=2.45522660545123,$$

$$b_{10}=0.868638616650473,b_{11}=3.28470648761428,b_{12}=6.03856264501091,$$

$$b_{13}=0.783242196623145,$$

$$c_{1}=-1205.47857134292,c_{2}=-0.0706957602179132, c_{3}=1.85940197461742,$$

$$c_{4}=0.156088498545416,c_{5}=0.0154923159762741,c_{6}=4.58410568976578,$$

$$c_{7}=8.20112285023355,c_{8}=0.0102445378264715,c_{9}=4.34280170411788,$$

$$c_{10}=0.573266617564107,c_{11}=0.0132611128090139,c_{12}=2.81049429460953,$$

$$c_{13}=9.78142032899542,$$

$$d_{1}=-22.3061204796155,d_{2}=0.491851654980196,d_{3}=3.60044697469808,$$

$$d_{4}=1.57318913997506,d_{5}=0.533973870966418,d_{6}=2.95238677712298,$$

$$d_{7}=0.398879464168394,$$

$$\alpha_{1}=7.16762374637002,\alpha_{2}=-2.29923908883952,\alpha_{3}=0.993610036976414,$$

$$\alpha_{4}=0.822653301898728,\alpha_{5}=0.351789069048571,\alpha_{6}=2.3433371713387,$$

$$\alpha_{7}=2.69518809936406,\alpha_{8}=8.23433806743924,\alpha_{9}=2.11387512100253,$$

$$\alpha_{10}=2.70320093145591,\alpha_{11}=1.34988673630637,\alpha_{12}=0.661864146442562$$

$$\begin{aligned} y=a_{1}\left( y_{1}+y_{2}-y_{3}-a_{2}-y_{4} \right)^{2}+y_{5}-42.9724593139663\#\left( A7 \right) \end{aligned}$$

$$\begin{aligned} y_{1}=a_{3}\sin\left( a_{4}x_{4}^{'}+a_{5} \right)\#\left( A8 \right) \end{aligned}$$

$$\begin{aligned} y_{2}=b_{1}\tan\left( b_{2}x_{1}^{'}+b_{3} \right)\#\left( A9 \right) \end{aligned}$$

$$\begin{aligned} y_{3}=\mathrm{atan} \left( c_{1}x_{3}^{'}-c_{2} \right)\#\left( A10 \right) \end{aligned}$$

$$\begin{aligned} y_{4}=d_{1}e^{-d_{2}\left( d_{3}-x_{2}^{'} \right)^{2}}\#\left( A11 \right) \end{aligned}$$

$$\begin{aligned} y_{5}=\alpha_{1}\sin\left( \alpha_{2}\left( \alpha_{3}-x_{1}^{'} \right)^{2}+\alpha_{4}\cos\left( \alpha_{5}x_{3}^{'}+\alpha_{6} \right)+\alpha_{7} \right)\#\left( A12 \right) \end{aligned}$$

**Eqs. (A7-A12)** represent the explicit formulas of the KAN model in the second iteration, where $x_{1}^{'},x_{2}^{'},x_{3}^{'},x_{4}^{'}$ correspond to the preprocessed reaction conditions (reaction time, catalyst concentration, proton addition ratio, and reaction temperature, respectively) after quantile transformation. $y$ denotes the H_2_O_2_ production.

$$a_{1}=123.054858425453,a_{2}=0.168477555109607,a_{3}=0.889442642796019,$$

$$a_{4}=5.31521509657421,a_{5}=3.62858430228827,$$

$$b_{1}=0.0727089724058087,b_{2}=1.51088225863682, b_{3}=8.42816525004492,$$

$$c_{1}=4.69735932923777,c_{2}=1.48433734148536,$$

$$d_{1}=0.685409231162558,d_{2}=6.74407115602818, d_{3}=0.67534007023462,$$

$$\alpha_{1}=42.0761057641326,\alpha_{2}=-13.3316814975314, \alpha_{3}=0.466046865870268,$$

$$\alpha_{4}=2.12354996999579,\alpha_{5}=6.1173796207218, \alpha_{6}=4.1340390806775,$$

$$\alpha_{7}=5.45270519469013$$

## **2.3 Expanded formula**

$$\begin{aligned} y=-6.92413831734146e^{9.10908297635869x_{2}^{'}}e^{-6.74407115602818{x_{2}^{'}}^{2}} \\ \sin\left( 5.31521509657421x_{4}^{'}+3.62858430228827 \right) \\ -0.566025236059029e^{9.10908297635869x_{2}^{'}}e^{-6.74407115602818{x_{2}^{'}}^{2}} \\ \tan\left( 1.51088225863682x_{1}^{'}+8.42816525004492 \right) \\ +7.78480588200156e^{9.10908297635869x_{2}^{'}}e^{-6.74407115602818{x_{2}^{'}}^{2}} \\ \mathrm{atan} \left( 4.69735932923777x_{3}^{'}-1.48433734148536 \right) \\ +1.31156506200251e^{9.10908297635869x_{2}^{'}}e^{-6.74407115602818{x_{2}^{'}}^{2}} \\ +0.123122328114253e^{18.2181659527174x_{2}^{'}}e^{-13.4881423120564{x_{2}^{'}}^{2}} \\ +97.3497093743761\sin\left( 5.31521509657421x_{4}^{'}+3.62858430228827 \right)^{2} \\ +15.9160287398955\sin\left( 5.31521509657421x_{4}^{'}+3.62858430228827 \right) \\ \tan\left( 1.51088225863682x_{1}^{'}+8.42816525004492 \right) \\ -218.90047697365\sin\left( 5.31521509657421x_{4}^{'}+3.62858430228827 \right) \\ \mathrm{atan} \left( 4.69735932923777x_{3}^{'}-1.48433734148536 \right) \\ -36.8798171728473\sin\left( 5.31521509657421x_{4}^{'}+3.62858430228827 \right) \\ +42.0761057641326\sin\left( \begin{aligned} -13.3316814975314{x_{1}^{'}}^{2}+12.4263767574103x_{1}^{'} \\ +2.12354996999579\cos\left( 6.1173796207218x_{3}^{'}+4.1340390806775 \right) \\ +2.55706822373302 \end{aligned} \right) \\ +0.650541158461476\tan\left( 1.51088225863682x_{1}^{'}+8.42816525004492 \right)^{2} \\ -17.8943846113139\tan\left( 1.51088225863682x_{1}^{'}+8.42816525004492 \right) \\ \mathrm{atan} \left( 4.69735932923777x_{3}^{'}-1.48433734148536 \right) \\ -3.01480216950514\tan\left( 1.51088225863682x_{1}^{'}+8.42816525004492 \right) \\ +123.054858425453\mathrm{atan}\left( 4.69735932923777x_{3}^{'}-1.48433734148536 \right)^{2} \\ +41.4639633837583\mathrm{atan}\left( 4.69735932923777x_{3}^{'}-1.48433734148536 \right)-39.4795857259414\#\left( A13 \right) \end{aligned}$$

**Eqs. A13** shows the expansion of **Eqs. (7-12)** in the second iteration, where $x_{1}^{'},x_{2}^{'},x_{3}^{'},x_{4}^{'}$ correspond to the preprocessed reaction conditions (reaction time, catalyst concentration, proton addition ratio, and reaction temperature, respectively) after quantile transformation. $y$ denotes the H_2_O_2_ production.

# **3 Application of BayesianKAN to flavone synthesis**

## **3.1 Quantile transformed data**

**Tab. S5** Reaction condition data after quantile transformation in the first iteration

| Eosin Y concentration | HClO_4_ concentration | Reaction time |
| --- | --- | --- |
| 0.230769 | 0.192308 | 0.230769 |
| 0.769231 | 0.192308 | 0.230769 |
| 0.230769 | 0.846154 | 0.230769 |
| 0.769231 | 0.846154 | 0.230769 |
| 0.230769 | 0.846154 | 0.769231 |
| 0.076923 | 0.576923 | 0.576923 |
| 0.923077 | 0.576923 | 0.576923 |
| 0.538462 | 0.076923 | 0.576923 |
| 0.538462 | 0.576923 | 0.076923 |
| 0.538462 | 0.576923 | 0.576923 |
| 0.692308 | 0.307692 | 0.923077 |
| 0.384615 | 0.384615 | 0.846154 |

**Tab. S6** Reaction condition data after quantile transformation in the second iteration

| Eosin Y concentration | HClO_4_ concentration | Reaction time |
| --- | --- | --- |
| 0.214286 | 0.214286 | 0.250000 |
| 0.821429 | 0.214286 | 0.250000 |
| 0.214286 | 0.857143 | 0.250000 |
| 0.821429 | 0.857143 | 0.250000 |
| 0.214286 | 0.857143 | 0.785714 |
| 0.071429 | 0.607143 | 0.535714 |
| 0.928571 | 0.607143 | 0.535714 |
| 0.500000 | 0.071429 | 0.535714 |
| 0.500000 | 0.607143 | 0.071429 |
| 0.500000 | 0.607143 | 0.535714 |
| 0.642857 | 0.357143 | 0.928571 |
| 0.357143 | 0.428571 | 0.857143 |
| 0.821429 | 0.214286 | 0.785714 |

**Tab. S7** Reaction condition data after quantile transformation in the third iteration

| Eosin Y concentration | HClO_4_ concentration | Reaction time |
| --- | --- | --- |
| 0.233333 | 0.233333 | 0.233333 |
| 0.800000 | 0.233333 | 0.233333 |
| 0.233333 | 0.866667 | 0.233333 |
| 0.800000 | 0.866667 | 0.233333 |
| 0.233333 | 0.866667 | 0.766667 |
| 0.066667 | 0.633333 | 0.500000 |
| 0.933333 | 0.633333 | 0.500000 |
| 0.533333 | 0.066667 | 0.500000 |
| 0.533333 | 0.633333 | 0.066667 |
| 0.533333 | 0.633333 | 0.500000 |
| 0.666667 | 0.400000 | 0.933333 |
| 0.400000 | 0.466667 | 0.866667 |
| 0.800000 | 0.233333 | 0.766667 |
| 0.233333 | 0.233333 | 0.766667 |

**Tab. S8** The value of the independent variable recommended by BayesianKAN in the three rounds of iteration (the value after quantile transformation)

|  | Eosin Y concentration | HClO_4_ concentration | Reaction time |
| --- | --- | --- | --- |
| 1^st^ iteration | 0.769231 | 0.192308 | 0.769231 |
| 2^nd^ iteration | 0.214286 | 0.214286 | 0.785714 |
| 3^rd^ iteration | 0.659259 | 0.964017 | 0.995264 |

## **3.2 Explicit formulas**

$$\begin{aligned} y=a_{1}-a_{2}\left( y_{1}+y_{2}-y_{3}-a_{3} \right)^{2}\#\left( A14 \right) \end{aligned}$$

$$\begin{aligned} y_{1}=\left( a_{4}-x_{2}^{'} \right)^{4}\#\left( A15 \right) \end{aligned}$$

$$\begin{aligned} y_{2}=a_{5}cos(a_{6}x_{1}^{'}+a_{7})\#\left( A16 \right) \end{aligned}$$

$$\begin{aligned} y_{3}=a_{8}\cos\left( a_{9}x_{3}^{'}+a_{10} \right)\#\left( A17 \right) \end{aligned}$$

**Eqs. (A14-A17)** represent the explicit formulas of the KAN model in the first iteration, where $x_{1}^{'},x_{2}^{'}{,x}_{3}^{'}$ correspond to the preprocessed reaction conditions (Eosin Y concentration (mol%), HClO_4_ concentration (equiv), reaction time (h)) after quantile transformation. $y$ denotes the flavone yield.

$$a_{1}=0.969677147373068,a_{2}=166.864121594478,a_{3}=0.0146592448209565,$$

$$a_{4}=0.526806499457233,a_{5}=0.0487913904004378,a_{6}=2.89992002145933,$$

$$a_{7}=7.17311921752583,a_{8}=0.0271550379328221,a_{9}=4.32948936611575,$$

$$a_{10}=2.16591133568959$$

$$\begin{aligned} y=a_{1}\cos\left( y_{1}+y_{2}+y_{3}+a_{2} \right)-a_{3}\#\left( A18 \right) \end{aligned}$$

$$\begin{aligned} y_{1}=a_{4}\left( a_{5}-x_{2}^{'} \right)^{4}\#\left( A19 \right) \end{aligned}$$

$$\begin{aligned} y_{2}=a_{6}\left| a_{7}x_{3}^{'}-a_{8} \right|^{2}\#\left( A20 \right) \end{aligned}$$

$$\begin{aligned} y_{3}=a_{9}atan(a_{10}x_{1}^{'}-a_{11})\#\left( A21 \right) \end{aligned}$$

**Eqs. (A18-A21)** represent the explicit formulas of the KAN model in the second iteration, where $x_{1}^{'},x_{2}^{'}{,x}_{3}^{'}$ correspond to the preprocessed reaction conditions (Eosin Y concentration (mol%), HClO_4_ concentration (equiv), reaction time (h)) after quantile transformation. $y$ denotes the flavone yield.

$$a_{1}=-1.67038268640722,a_{2}=8.98173669966929,$$

$$a_{3}=0.689386738014389,a_{4}=-13.3744446947351,$$

$$a_{5}=0.537406163762317,a_{6}=0.156150461955979,$$

$$a_{7}=9.96856490797993,a_{8}=2.0179600852049,$$

$$a_{9}=0.437250755702091,a_{10}=4.81731106154287,$$

$$a_{11}=3.95040739489597$$

$$\begin{aligned} y=a_{1}-a_{2}\left( y_{1}+y_{2}-y_{3}+a_{3} \right)^{2}\#\left( A22 \right) \end{aligned}$$

$$\begin{aligned} y_{1}=a_{4}\left( a_{5}-x_{1}^{'} \right)^{2}\#\left( A23 \right) \end{aligned}$$

$$\begin{aligned} y_{2}=\left( a_{6}-x_{2}^{'} \right)^{2}\#\left( A24 \right) \end{aligned}$$

$$\begin{aligned} y_{3}=a_{7}\cos\left( a_{8}x_{3}^{'}+a_{9} \right)\#\left( A25 \right) \end{aligned}$$

**Eqs. (A22-A25)** represent the explicit formulas of the KAN model in the third iteration, where $x_{1}^{'},x_{2}^{'}{,x}_{3}^{'}$ correspond to the preprocessed reaction conditions (Eosin Y concentration (mol%), HClO_4_ concentration (equiv), reaction time (h)) after quantile transformation. $y$ denotes the flavone yield.

$$a_{1}=0.96997949843601,a_{2}=5.22263554315609,a_{3}=0.142910887617524,$$

$$a_{4}=-0.747946054684404,a_{5}=0.275838451883883,a_{6}=0.552640739032443,$$

$$a_{7}=0.167646054531505,a_{8}=4.35052006738205,a_{9}=8.58372407046943$$

## **3.3 Expanded formula**

$$\begin{aligned} y=-2.92166401399934{x_{1}^{'}}^{4}+3.22362911418572{x_{1}^{'}}^{3}+7.81249929911628{x_{1}^{'}}^{2}{x_{2}^{'}}^{2} \\ -8.63501077270812{x_{1}^{'}}^{2}x_{2}^{'} \\ -1.30973468352699{x_{1}^{'}}^{2}\cos\left( 4.35052006738205x_{3}^{'}+8.58372407046943 \right) \\ +2.16871928038205{x_{1}^{'}}^{2}-4.30997542402431x_{1}^{'}{x_{2}^{'}}^{2} \\ +4.76373600708892x_{1}^{'}x_{2}^{'} \\ +0.722550374965426x_{1}^{'}\cos\left( 4.35052006738205x_{3}^{'}+8.58372407046943 \right) \\ -1.68698391738992x_{1}^{'}-5.22263554315609{x_{2}^{'}}^{4}+11.5449646650675{x_{2}^{'}}^{3} \\ +1.75110848613224{x_{2}^{'}}^{2}\cos\left( 4.35052006738205x_{3}^{'}+8.58372407046943 \right) \\ -10.4686411949499{x_{2}^{'}}^{2} \\ -1.93546777580421x_{2}^{'}\cos\left( 4.35052006738205x_{3}^{'}+8.58372407046943 \right) \\ +4.51885864783391x_{2}^{'} \\ -0.146783214378354\cos\left( 4.35052006738205x_{3}^{'}+8.58372407046943 \right)^{2} \\ +0.685408050645406\cos\left( 4.35052006738205x_{3}^{'}+8.58372407046943 \right) \\ +0.169846802951834\#\left( A26 \right) \end{aligned}$$

**Eqs. A26** shows the expansion of **Eqs. (13-16)** in the third iteration, where $x_{1}^{'},x_{2}^{'}{,x}_{3}^{'}$ correspond to the preprocessed reaction conditions (Eosin Y concentration (mol%), HClO_4_ concentration (equiv), reaction time (h)) after quantile transformation. $y$ denotes the flavone yield.
